# Real-world usage of mass rapid antigen testing for COVID-19 in long-term care facilities and support programmes: results from long-term surveillance in North-Eastern Germany

**DOI:** 10.1186/s12889-025-22914-x

**Published:** 2025-05-15

**Authors:** Tillmann Görig, Josefin Pauline Haß, Anastasia Tavakina, Vivien Giermann, Sebastian Karaytug, Nils-Olaf Hübner

**Affiliations:** Central Unit for Infection Prevention and Control, Institute of Hygiene and Environmental Medicine, University Medicine Greifswald, Walther-Rathenau-Str. 49a, 17475 Greifswald, Germany

**Keywords:** Public health, Prevention, Infections, COVID-19, Mass screening, Point-of-care testing

## Abstract

**Background:**

From December 2020 to February 2023, the research project ZEPOCTS operated as a central surveillance centre for COVID-19 rapid antigen tests (RATs) in the German state Mecklenburg-Western Pomerania (M-W). Since mid-December 2020, long-term care facilities (LTCF) as well as support programmes in M-W had been obliged by ordinance to report on-site RATs to this surveillance project. However, most studies have measured RATs in cross-sectional studies or short-term comparisons with smaller samples, and only a few studies have followed the long-term development of COVID-19 testing, even though the pandemic lasted more than two years. The aim of this article is to present the surveillance methods and provide an overview of the outcome development of the results of RATs in LTCF and support programmes as well as a comparison with the infection development of the pandemic.

**Methods:**

The project was designed as a prospective longitudinal surveillance study. The analysis includes around 6,2 million RATs of 1,015 facilities for 120 weeks. For comparative analysis of the RATs’ development in the LTCF and regional development of the pandemic, several inferential correlation tests and a nonparametric multiple changepoint detection analysis with pruned exact linear time (PELT) and changepoints over a range of penalties (CROPS) were performed.

**Results:**

The results indicate that the weekly positivity rates of RATs and polymerase chain reaction (PCR) tests correlated highly. The changepoint analysis revealed that changepoints of increase are primarily found earlier in the PCR distribution. Both the use of RATs by inpatient long-term care facilities and the distribution of the positivity rate of support programmes differed significantly from the other categories.

**Conclusions:**

The study demonstrated a delayed increase in the RATs positivity rate in the participating facilities compared to PCR positivity rate of public health data. Still, it was observed that the positivity rate of RATs evidently follows the pandemic dynamics. We conclude that a frequent large-scale testing strategy was feasible but should consider reasonable adjustments to preserve existing resources. Further research is necessary to identify improvements for future applications.

**Supplementary Information:**

The online version contains supplementary material available at 10.1186/s12889-025-22914-x

## Introduction

The COVID-19-pandemic has been a global public health challenge. At the beginning of the pandemic fast and simple solutions for large-scale testing were scarce. Despite being the gold standard to diagnose SARS-CoV-2, polymerase chain reaction (PCR) testing was limited in its scale by its costs, logistics, turn-around times, and need for skilled personnel [1–8]. As part of the pandemic response, public health authorities as well as several researchers advocated for a frequent large-scale rapid testing (FLSRT) strategy. This strategy aimed at the early detection of active infections [1–6, 9–13]. With the development of rapid antigen tests (RATs) for SARS-CoV-2, large-scale testing of asymptomatic individuals became feasible. Furthermore, the broad preventive screening using RATs for FLSRT was associated with expectations that infected individuals could be early identified and isolated, transmission chains interrupted and lockdown measures eased [2, 6, 10, 14]. To date, real-world evaluations of FLSRT strategies for asymptomatic individuals are still rare but would be much needed, especially in health care facilities, taking into account clinical and economic implications [4, 15–17]. Evaluations of real-world usage are complicated since in most countries and settings RATs were introduced and widely distributed without an accompanying field research strategy. However, at the end of 2020, the state government of the North-Eastern German federal state Mecklenburg-Western Pomerania (M-W) imposed the first legal obligations to report test results of RATs conducted in inpatient and outpatient long-term care facilities (LTCF) as well as support programmes. Simultaneously it commissioned the research project ZEPOCTS (“Zentrale Erfassung von COVID-19 Antigen-Schnelltests”– central surveillance of COVID-19 antigen tests) to collect, monitor, and evaluate their usage of RATs. Therefore, in addition to the question of feasibility in regular operation, in terms of frequency of applications and their test results as well as the usability of the result display, the aspect of the type of facility also had to be taken into account. Furthermore, the ongoing use and the development of results compared to the pandemic situation needed to be evaluated. Here, we present a first overview of the ZEPOCTS surveillance comparing the frequency of results and the development of RATs by facility category as well as comparing the RATs’ development with official PCR results as an indicator for the dynamics of the COVID-19-pandemic.

## Methods

As described above, ZEPOCTS was set up by ordinance of the government of M-W to collect data on RATs from care facilities for vulnerable groups. The University Medicine Greifswald (UMG) was commissioned with its implementation as part of the cooperative study “schugi-MV”, which was a collaboration with the Institute of Community Medicine of the UMG and the Department of Tropical Medicine and Infectious Diseases of the University Medicine Rostock. ZEPOCTS was planned as a prospective longitudinal surveillance study.

Data was received by calendar week from reportable facilities and collected from ISO week 47 of 2020 to week 9 of 2023. Initially e-mailed PDF questionnaires for data collection were replaced by an online reporting system (Remark Web Survey, Gravic Inc, 2021) in April 2021. Collected data included the total number of RATs and its results conducted by each group of tested people (residents/patients, personnel, visitors, and others) as well as information regarding the facility type, its district, and status of the implemented test strategy. The test results were recorded as *negative*, *positive*, and *invalid;* whereas *invalid* meant the test was not analysable or users got no clear result. Facilities were also encouraged to report results of confirmatory PCR tests for positive RATs. Facility type included full or partial inpatient and outpatient LTCF, services for patients and people with disabilities as well as sheltered workshops, shared accommodations, and day groups. Depending on these types, characteristics and the pandemic status, facilities were obligated to test at different frequencies. Facility types were classified into four main categories: *inpatient LTCF*, *outpatient LTCF*, *support programmes*, and *shared accommodations* (Supplementary Table 1).

Statistical analysis was conducted using R 4.1.3 [18]. Figures were created using the package ggplot2 [19]. To assess the use and development of RATs over time in general and by type of facility, the following analysis uses the absolute and relative numbers of total, negative, positive and non-evaluable test results. Several different statistical methods were used to analyse the different aspects. Correlation tests were carried out to compare the RAT utilisation and positivity rate data between the different facilities. Therefore the analysis included the Kruskal-Wallis test to examine the corresponding data on the number of rapid tests performed and the proportion of positive test results.

For a more detailed comparative analysis of the development of RATs over time, PCR testing was used as the only other indicator for SARS-CoV-2 infections available to us. In the comparison of the RAT and PCR results, the Pearson’s r correlation test was used in the first step. In general, there is a high correlation between RATs and PCR testing, however this correlation does not consider time as a factor. In order to explore how RATs followed pandemic dynamics and to evaluate the temporal performance of the RAT positivity rates, the time series of the PCR and RAT positivity rates were compared with each other using a changepoint analysis. Since both PCR and RAT results are only proxies of an undetectable true empirical infection status, a measurable true independent variable, the real infection, is missing. This leads to the challenge of comparing two quasi-dependent variables without a regressor. Causal analyses such as multivariate regression in a time-series-cross section analysis are therefore not applicable. We therefore decided to use changepoint analysis to analyse the temporal performance of both test results. The SARS-CoV-2 infection waves are officially defined by the federal PCR results, therefore we have to assess the RATs’ timely development and performance in comparison to the official PCR results and therefore official infection phases. To compare the surveillance RATs data with the dynamics of the pandemic, official PCR testing data for the study period of the entire population of the federal state M-W was kindly provided by the State Authority for Health and Social Affairs (*Landesamt für Gesundheit und Soziales*, *LAGuS*). In Germany, only PCR-confirmed infections are officially counted as cases [20]. Definitions of individual COVID-19 waves in Germany were adopted from the Robert Koch Institute (RKI) [21]. The data given on official PCR tests therefore provide the best possible picture of the true infection situation in this study area.

For comparative analysis of the weekly positivity rates of RATs and PCR tests, we used a nonparametric multiple changepoint detection analysis (NMCD), as proposed by Haynes and colleagues [22, 23], and based on the work of Zou and colleagues [24]. NMCD is a method that detects changepoints where characteristics of a dataset, like the mean or variance, shift significantly, without presuming a specific distribution. When a potential changepoint is found, the analysis tests whether the difference is random or significant. The NMCD algorithm evaluates the given data using a maximum log-likelihood function as a segment cost function model to identify possible changepoints in the empirical distribution. The distribution is divided into cost segments. Optimal segmentation is calculated with a minimised penalty cost function, to find the best combination of changepoints. The used algorithm is pruned exact linear time (PELT) [25]. For a more detailed explanation of the changepoint analysis and methodological details such as PELT please also see the Supplementary information. The penalty is required to avoid under and over-fitting by penalising the addition of new changepoints and therefore optimising the quality of the detected changepoints. We used a penalty range of min = 2 to max = 2*2log(n) for the z-transformed positivity rates in 120 weeks. One advantage of the applied method is the additional use of changepoints over a range of penalties, called CROPS and the *elbow* interpretation method to identify the optimal number of changepoints [26]. In summary, *CROPS* is an extension of PELT that examines a range of penalties within the NMCD framework and discovers the most robust and stable changepoints.

**Fig. 1:**
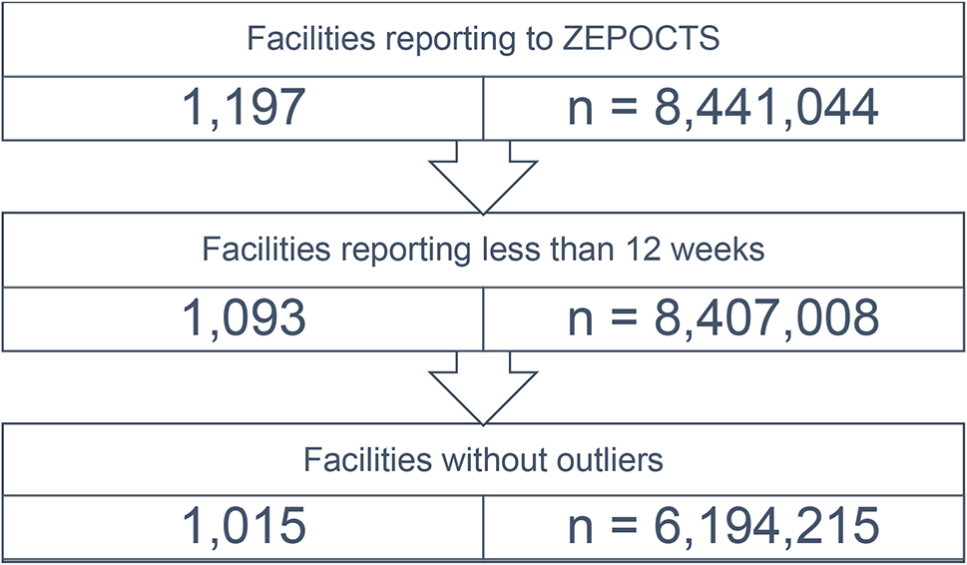
Flowchart of facilities reporting to ZEPOCTS

In order to guarantee continuity of the test system within the facilities for at least a quarter of a year, facilities reporting less than 12 weeks were excluded from the analysis, resulting in a figure of 1,093 from the previous 1,197 facilities (Fig. 1). This filtering had no significant impact on the results of the analysis. The 1,093 facilities reported 8,407,008 RATs in total. Outliers were identified by the Tukey fence method and further 78 facilities were suspended [27].

## Results

The following analysis includes 1,015 facilities with a total of 6,194,215 RATs (for more information see Supplementary Tables 2 and Supplementary Fig. 1).

### Total and weekly usage of RATs

**Fig. 2:**
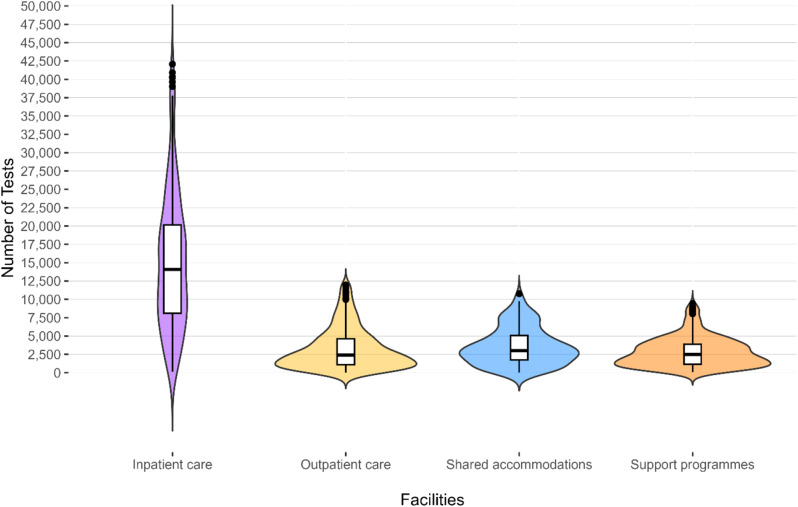
Distribution of number of RATs by facility category, without outliers

**Fig. 3:**
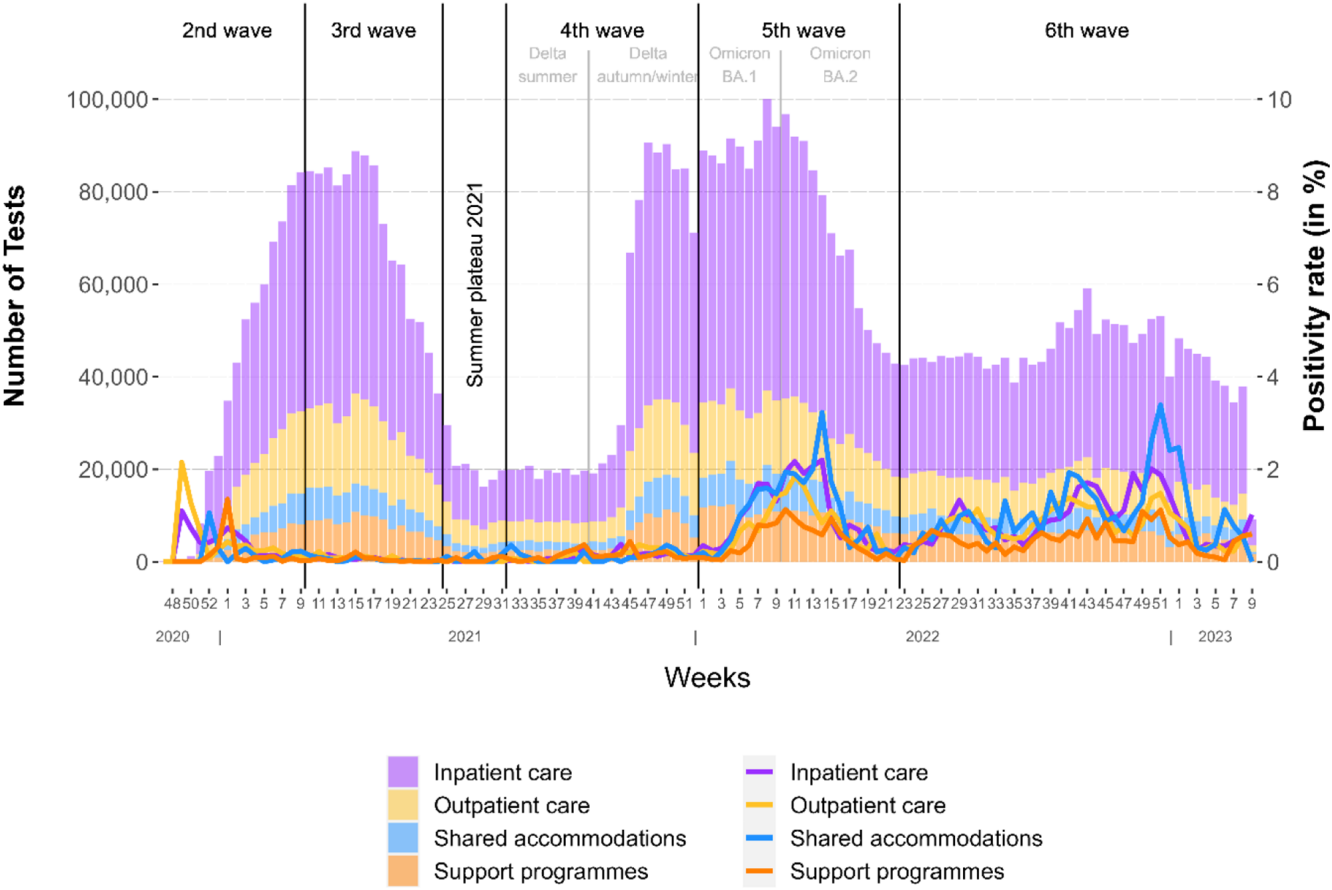
Number and positivity rate of RATs by facility category and ISO week. Number and positivity rate from week 47 of 2020 to week 9 of 2023, with classification of COVID-19-waves in Germany by the RKI [21]

The usage of RATs was significantly different between *inpatient LTCF* and the other categories (Kruskal-Wallis chi²=387.4, *p* < 0.01) (Fig. 2). *Inpatient* and *outpatient LTCF* were the main users of RATs. *Inpatient LTCF* exhibited the highest mean utilization rate, followed by *outpatient LTCF* and s*hared accommodations*, and ending with *support programmes* (Table 1). Due to the second and third COVID-19 waves, the beginning of 2021 was marked by high testing frequency (Fig. 3). During summer 2021, rapid antigen positivity and testing were low compared to the rest of the distribution. With the upcoming autumn testing increased again due to the fourth infection wave. The usage of RATs finally reached its peak during the fifth wave in 2022.

**Table 1:** Results of COVID-19 rapid antigen testing by facility category

Facility category (*n*)	Total (2020–2023^a^)						Weekly		
	Reported weeks (total)	Total number of RATs used	Average number of reported RATs used (CI 95) per facility	Rate of negative RATs (*n*)	Rate of positive RATs (*n*)	Rate of invalid RATs (*n*)	Average number of reported RATs used (CI 95) per facility	SD	Median
*Inpatient long-time care facilities (247)*	120	3,762,514	15,232.85 (14,052.1–16,413.6)	99.21 (3,732,761)	0.64 (24,020)	0.15 (5,733)	174.21 (160.76–187.67)	107.36	155.88
*Outpatient long-time care services (360)*	120	1,182,201	3,283.89 (2,990.71–3,577.07)	99.32 (1,174,220)	0.49 (5,743)	0.19 (2,238)	45.95 (41.6–50.3)	41.99	33.6
*Shared accommodations (143)*	117	506,963	3,545.2 (3,126.66–3,963.73)	99.07 (502,242)	0.7 (3,532)	0.23 (1,189)	42.65 (36.88–48.42)	34.91	35.67
*Support programmes (265)*	120	742,537	2,802.03 (2,561.45–3,042.6)	99.5 (738,857)	0.33 (2,441)	0.17 (1,239)	38.49 (34.63–42.36)	31.94	31.98
***Total*** *(1015)*	120	6,194,215	6,102.67 (5,653.46–6,551.89)	99.26 (6,148,080)	0.58 (35,736)	0.17 (10,399)	74.75 (69.58–79.92)	83.96	43.73

Abbreviations: CI 95 = confidence interval 95%, RATs = rapid antigen tests, SD = standard deviation. Legend: ^a^ ISO week 47 of 2020 to ISO week 9 of 2023

### Total and weekly rate of positive and negative results

Overall, most RATs were reported as negative, whereas the overall positivity rate of RATs and rate of invalid tests was relatively low. S*hared accommodations* showed the highest overall positivity rate, while *inpatient* and *outpatient LTCF* experienced lower overall positivity rates. *Support programmes* had the lowest overall positivity rate (Table 1). Correlation tests show the distribution of positivity rates of *support programmes* differs significantly from the positivity rate of the other categories (Kruskal-Wallis chi²=86.95, *p* < 0.01).

**Fig. 4:**
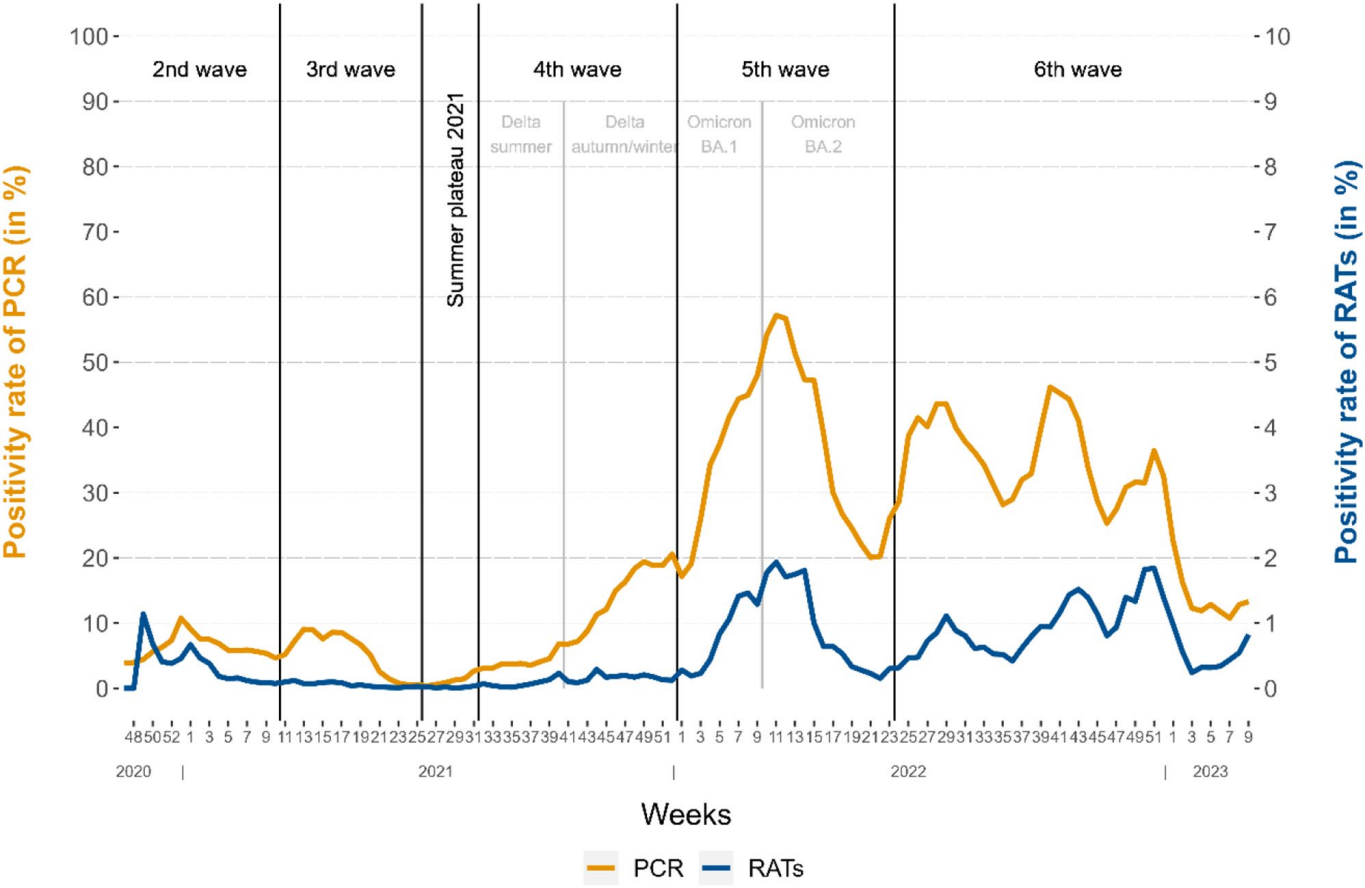
Positivity rate of PCR and rapid antigen testing in M-W by ISO week. Positive rates from week 47 of 2020 to week 9 of 2023, with classification of the COVID-19-waves in Germany by the RKI [21]. The LAGuS reported a total of 3,076,782 PCR tests for this period

Besides the initial weeks in 2020, the minimum positivity rate was recorded in the summer of 2021 (week 29) with 0.006% (*n* = 1 of 16,255). A noticeable spike in the positivity rates of RATs occurred when Omicron-variants became dominant in the beginning of 2022 [28]: the maximum weekly positivity rate reached 1.9% in week 11 of 2022 (*n* = 1,778 of 91,888). Comparing peaks, *inpatient LTCF* reached their highest weekly positivity rate in week 14 of 2022 at 2.2% (*n* = 1,028 of 46,642), while *outpatient LTCF*, apart from the initial weeks, reached their highest positivity rate earlier in week 11 of 2022 at 1.84% (*n* = 307 of 16,658) (Fig. 3). Of the total positive RATs reported to ZEPOCTS, 16.9% (*n* = 6,050) were verified by PCR and reported by the facilities. Of these, 5,309 (87.8%) were confirmed by positive PCR results.

### Total and weekly rate of invalid results

The visualisation of the invalidity rate reveals a certain dynamic, which, however, does not show any major differences over time and between the types of facilities. It ranges between 0% and 1.16%. (Supplementary Fig. 2). Apart from the initial weeks in 2020 (week 51: 1.16%, *n* = 97 of 8,341) the peak of the weekly overall invalidity rate was observed early in week 3 in 2021 with 0.34% (*n* = 178 of 52,395). The facility category with the highest overall invalidity rate is s*hared accommodations*, while most invalid tests occurred in *inpatient LTCF* (Table 1).

### Comparison of RAT and PCR weekly positivity rate

During the study period (120 weeks), a total of 3,076,782 officially reported PCR tests were performed in the study area, resulting in 724,098 positive tests (positivity rate: 23.53%). The weekly positivity rate of RATs correlated highly with the positivity rate of PCR tests (Pearson’s *r* = 0.826, *p* < 0.01) (Fig. 4).

### Changepoint analysis of weekly positivity rates

**Fig. 5:**
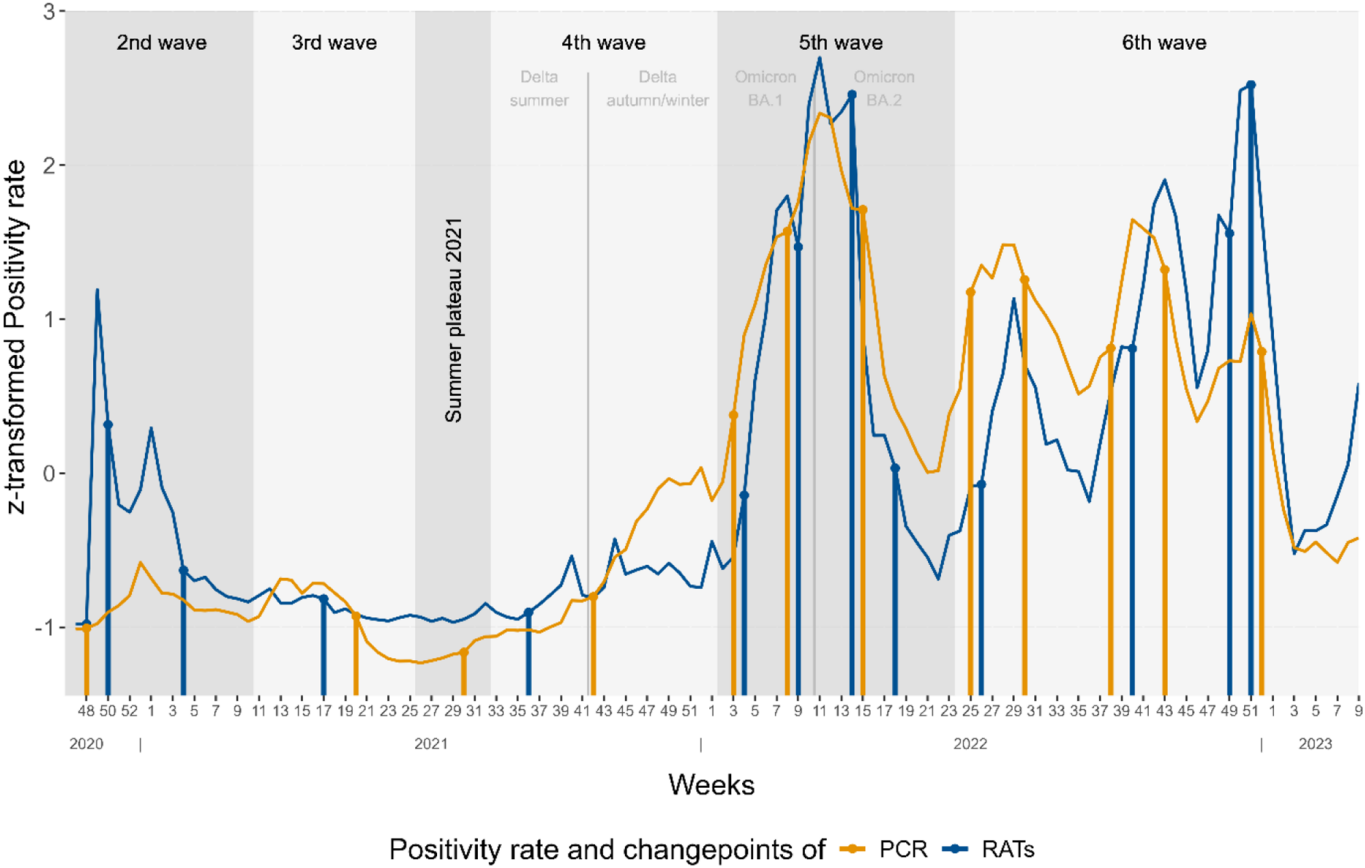
Changepoints of the z-transformed positivity rate of PCR and RATs distribution by ISO week. Changepoints from week 47 of 2020 to week 9 of 2023, with classification of COVID-19-waves by the RKI [21]

For a comparative analysis of the weekly positivity rates of PCR tests and RATs, we choose the NMCD to compare z-transformed rate values. After applying the *CROPS* and *elbow* method, 13 changepoints are selected as the optimum number for RATs and 12 changepoints for PCR tests distribution (Supplementary Fig. 3). These numbers of changepoints sufficiently explain the distributions and additional points only marginally increase the explanatory power. The changepoints of both distributions are shown in Fig. 5. At the end of 2020, both distributions of positivity rate show changepoints of increase. The following first half of 2021 is characterized by decreasing elements. In the summer of 2021 the distribution of PCR tests is already demonstrating an increase, which is followed by the distribution of RATs, six weeks later during the fourth wave. The rise continues in 2022 and culminates at high levels in the fifth wave of the Omicron-variants. After a break in spring 2022, both distributions show an increasing momentum in the summer. The subsequent development is characterised by an alternating pattern of the positivity rate. Changepoints of increase are mostly found earlier in the distribution of PCR tests than in the distribution of RATs. And vice versa changepoints of decrease largely appeared earlier in the distribution of RATs. For further comparison, graphs of the differences in weekly numbers and positivity rates of the usage of PCR tests and RATs are shown in the supplementary document (Supplementary Fig. 4, Fig. 5).

## Discussion

In fall 2020, RATs were introduced to enable rapid, simple, low cost, and therefore widely applicable testing for SARS-CoV-2. During the pandemic, they were used in many countries for broad screening of asymptomatic individuals [2, 10]. Here, we present data on the usage of RATs for preventive screening in LTCF and support facilities in North Eastern Germany over 120 weeks from the ZEPOCTS project, one of the largest databases on real-world usage of RATs known to us.

Our data shows that RATs were used as a high-frequent, large-scale testing instrument in LTCF and support programmes. Main users were LTCF, especially inpatient care facilities. The use and positivity rates of RATs differed significantly between the facility categories. Although *inpatient LTCF* showed a significantly higher use than other categories, a significantly lower overall positivity rate occurred in *support programmes*. While the former is most likely attributable to facility characteristics, testing needs, and the legal regulations and obligations, the latter is ambiguous and requires further investigation.The findings of lower positivity rate suggest two possible assumptions with regard to accommodation. Either the facility types of *inpatient care* and *shared accommodations* harbour a higher risk of infection, or the facility types *outpatient care* and *support programmes* and their associated test systems identify infection less fequently. This could potentially be due to poorer handling of the testing material, which affects reliability and thus accuracy [29].

The comparison of the values of the overall positivity rates of RATs and PCR demonstrates a clear distinction. The comparatively low positivity rate of RATs could be explained by their frequent use as a preventive measurement for asymptomatic individuals in facilities with enhanced infection control measures. Those individuals had a low pre-test probability to be positive, while PCR tests were typically used for individuals with symptoms or contact with confirmed cases.

However, the weekly positivity rate of RATs correlated highly with the positivity rate of PCR tests. A link between results of RATs and pandemic development in the research area can be seen from results of the changepoint analysis. Both positivity rate distributions show a similar number and distribution of optimal changepoints and a very symmetrical movement over time. The positivity rate of PCR tests shows a consistently earlier appearance of changepoints of increase than in the distribution of RATs, which does not have large intervals. This can be interpreted in different ways. On the one hand, this could mean that RATs detected the COVID-19-waves later. On the other hand, it could mean that the waves consistently occurred later in the analysed facilities, which might illustrate the effectiveness of other protective measures in these facilities. However, the time differences are not substantial.

The usage of RATs showed a satisfactory performance in terms of readability and invalidity rate. The rate of invalid RATs was approximately a third of the positivity rate with noticeable differences over time and facility category. This could indicate handling problems, particularly in the beginning of testing as well as difficulties with residents/patients in facilities such as *LTCF*. This might be shown in the data from the initial weeks of the observed FLSRT strategy for the positivity and invalidity rate. Additionally, it is very likely that product quality and reliability were unstable in early charges [30].

Although direct PCR verification of RATs results was not a core task of ZEPOCTS, our PCR results are in line with the findings of other, topic-related studies [6, 12, 13, 31, 32]. For example, a Cochrane review in 2022 argued that RATs could be a reasonable addition to the laboratory PCR testing of symptomatic individuals [17]. Given that each false-positive RAT has a significant impact, particularly in the LTCF, potential benefits and harms of a RATs-based test strategy must be carefully weighed.

## Limitations

Due to the length of the study period and lack of ressources, several uncontrollable limiting influences on the validity of our results occurred. The facilities used various products with different quality, but which were all marketable in Germany at the time. Additionally, it was not mandatory for the facilities to report PCR results of positive RATs. Likewise, negative RATs were not checked by PCR tests, therefore statements on sensitivity and specificity of RATs are not possible from our data. Consequently, the validity of the RATs’ results data cannot be determined. But, given the large number of RATs collected, the margin of error is approaching zero compared to general COVID-19 rapid testing. An assumption of a generally low false-positive rate is supported by the findings of other studies on large-scale implementation of RAT testing [29, 31]. Nevertheless, research shows a decreasing average incubation period of SARS-CoV-2 in comparison with newer variants, which suggests that earlier and higher viral load and symptoms would be advantageous for RATs strategies [29, 33]. In contrast, laboratory evidence suggests that later variants, such as Omicron, are more difficult to detect as the tests require a higher viral load for detection [34]. Furthermore, the legal conditions and foundations for usage of RATs changed over time. For example, the obligatory frequency of use, especially of vaccinated or asymptomatic personnel, often varied over time and type of facility. This could have influenced the frequency of RATs per facility type and therefore the shown results.

## Conclusion

In conclusion, our results show that an FLSRT strategy was implemented with high performance figures over a period of 120 weeks. The analysis shows significant differences in the overall use of RATs and in the positivity rates by facility category, which should be most likely attributed to the legal requirements and the characteristics of the different facility types. Yet, the differences found cannot be explained by the data respectively the results themselves.

The applied FLSRT strategy showed adequate coverage of pandemic dynamics, a relatively low rate of invalid test results and a discernible difference in positivity rates between the different facility categories. In conjunction with other research, our results show that an implementation of an FLSRT strategy leads to a massive, but feasible, application of resources with multiple and long periods in times of low incidence and therefore of very low positivity rates. Therefore, we conclude the optimal use of rapid antigen testing for an FLSRT strategy as an surveillance system should be based on previously acquired knowledge and patterns in order to protect and preserve existing resources. The use of an FLSRT strategy and its scope should focus on and adapt to known factors like seasonal or regional waves of infection, and factors associated to vulnerability. An optimal implementation of an FLSRT strategy could be a useful addition to the clinical diagnosis, screening, and surveillance of infection waves. An implementation without those factors should only be considered when taking the available resources into account. These resources include personnel, financial, infrastructural, and characteristic features and capabilities of the health care facilities and health care system concerned. Further research into the performance and benefits of RATs is urgently needed. Differences in utilisation and performance between different types of medical and social care facility types and groups of subjects should be investigated. In particular, the effectiveness and increased efficiency of RAT strategies in epidemic scenarios in different institutions and different groups of people should be focussed on. In addition to analysing transmission risks and their prevention through RATs, specific cost-benefit analyses could also be carried out on the aspects of personnel, financial and spatial resources in different types of facilities when using FLSRT.

## Electronic supplementary material

Below is the link to the electronic supplementary material.

Supplementary Material 1

## Data Availability

Data for this analysis was collected with and accessed within the data environment of the UMG, except the PCR data provided by the LAGuS. Because of the legal obligations, the authors are unable to share the underlying single-facility level data. Code scripts, data dictionary, copies of data survey templates, a copy of the protocol, and pseudonymised or aggregated data will be provided by the corresponding author upon reasonable request (zepocts@med.uni-greifswald.de).

## Abbreviations

CI: Confidence interval
COVID-19: Coronavirus disease 2019
CROPS: Changepoints over a range of penalties
ISO: International Organization for Standardization
FLSRT: Frequent large-scale rapid testing
LTCF: Long-term care facilities
M-W: Mecklenburg-Western Pomerania
NMCD: Nonparametric multiple changepoint detection analysis
PELT: Pruned exact linear time
PCR: Polymerase chain reaction
RATs: Rapid antigen tests
RKI: Robert Koch Institute
SARS-CoV-2: Severe acute respiratory syndrome coronavirus 2
SD: Standard deviation
UMG: University Medicine greifswald
ZEPOCTS: “Zentrale Erfassung von COVID-19 Antigen-Schnelltests”– central surveillance of COVID-19 antigen tests.
