# Supplementary material for: Real-world usage of mass rapid antigen testing for COVID-19 in long-term care facilities and support programmes: results from long-term surveillance in North-Eastern Germany
Source: BMC Public Health. 2025 May 15;25:1785. doi: 10.1186/s12889-025-22914-x (PMC12080137; doi:10.1186/s12889-025-22914-x)
Supplement: Supplementary file 1 — Supplementary Material 1 [file 12889_2025_22914_MOESM1_ESM.docx]

**Supplemental Material**

**Real-World Usage of Mass Rapid Antigen Testing for COVID-19 in Long-Term Care Facilities and Support Programmes: Results from Long-Term Surveillance in North-Eastern Germany**

BMC Public Health

Tillmann Görig^1^*, Josefin Pauline Haß^1^*, Anastasia Tavakina^1^, Vivien Giermann^1^, Sebastian Karaytug^1^, Nils-Olaf Hübner^1^

**Affiliations:**

^1^ Central Unit for Infection Prevention and Control, Institute of Hygiene and Environmental Medicine, University Medicine Greifswald, Greifswald, Germany

* These authors contributed equally to this work and share first authorship.

**Correspondence:** Tillmann Görig (tillmann.goerig@med.uni-greifswald.de)

Walther-Rathenau-Str. 49a

17475 Greifswald

Germany

**Table of Content**

[Methods 3](#_Toc194326173)

[Changepoint detection analysis and PELT 3](#_Toc194326174)

[CROPS and elbow method 3](#_Toc194326175)

[Figures 4](#_Toc194326176)

[Tables 8](#_Toc194326177)

**List of Figures**

[Supplemental Fig. 1 Distribution of number of RATs by facility category with Tukey’s fence-outliers. 4](#_Toc194326182)

[Supplemental Fig. 2 Number and rate of invalid RATs by facility category and ISO week from week 47 2020 to week 9 2023, with classification of COVID-19-waves in Germany by the RKI (Tolksdorf et al. 2021). 5](#_Toc194326183)

[Supplemental Fig. 3. CROPS diagnostic of RATs and PCR cost degree of number of changepoints. 5](#_Toc194326184)

[Supplemental Fig. 4 Weekly number differences of PCR and rapid antigen testing by ISO week from week 47 2020 to week 9 2023, with classification of COVID-19-waves in Germany by the RKI (Tolksdorf et al. 2021). 6](#_Toc194326185)

[Supplemental Fig. 5 Weekly differences of the positivity rate of PCR and rapid antigen testing by ISO week from week 47 2020 to week 9 2023, with classification of COVID-19-waves in Germany by the RKI (Tolksdorf et al. 2021). 7](file:///S:\IHU_AG%20MRE\09_SCHUGI%20und%20Pflege\20_Publikation\Paper%201\07_Dokumente%20BMCPublicHealth\20250331_ZEPOCTS_Supplemental_Information.docx#_Toc194326186)

**List of Tables**

[Supplemental Table 1. Categories of COVID-19 rapid antigen testing reporting facilities. 8](#_Toc194326191)

[Supplemental Table 2. Results of COVID-19 rapid antigen testing by facility category. (Abbreviations: CI 95=confidence interval 95 %, SD=standard deviation) 9](#_Toc194326192)

# Methods

## Changepoint detection analysis and PELT

Pruned exact linear time (PELT) is an efficient algorithm used for changepoint detection analysis. It finds the optimal set of changepoints while minimizing the computational cost and thereby the number of combinations. In the first step, the entire dataset is processed as one segment. The algorithm then calculates a cost function for each possible changepoint within the segment. Subsequently, it compares the cost, also known as the penalty term, of adding a changepoint to the cost of not adding one. If adding a changepoint reduces the overall cost, PELT splits the segment at that point. The process is repeated for each additional computed segment until no further improvements are found. This is where PELT comes in to efficiently rejecting potential changepoints that are unlikely to be part of the optimal solution. This step reduces the computational effort and therefore makes PELT a fast and efficient method for recognising changepoints.

## CROPS and elbow method

CROPS, changepoints over a range of penalties, is an extension of the PELT algorithm that allows for more flexibility in detecting changepoints. In the PELT algorithm, the penalty term mentioned above is used to control the number of changepoints detected. The higher the penalty, the fewer changepoints are identified. However, choosing the right penalty value can be difficult. Instead of using a single penalty value, CROPS explores a range of penalties. It runs the PELT algorithm multiple times, each time with a different penalty value within the specified range. By doing this, CROPS generates a set of potential changepoint configurations. Each configuration corresponds to a different penalty level and represents a trade-off between the number of changepoints and the fit to the data. CROPS therefore enhances the NMCD approach by providing a way to explore different penalty levels and to identify the most suitable changepoint configuration. It allows to assess the sensitivity of the results to the choice of penalty and select the most appropriate set of changepoints based on their specific needs.

The elbow interpretation method is a way to choose the optimal penalty value when using PELT and CROPS for changepoint detection. When running CROPS with a range of penalty values, it will identify multiple changepoint configurations. Each configuration has a different number of changepoints and a corresponding cost function value. The ‘elbow’ interpretation method then plots the cost for each configuration against the number of changepoints. On the plot, the curve starts to steep and then flattens out as the number of changepoints increases.

The so-called ‘elbow’ is the point at which the curve begins to level off. It therefore presents a balance between the number of changepoints and the fit to the data. The choice of the penalty value, which corresponds to the ‘elbow’, thus offers a good compromise between model complexity, in the form of the number of change points, and data fit. By using the ‘elbow’ interpretation method a balance between underfitting, which means too few changepoints, and overfitting, which means too many changepoints, is to be found. The method supports the choice of a penalty value that captures the most significant changes in the data without being too sensitive to noise.

# Figures

Supplemental Fig. 1 Distribution of number of RATs by facility category with Tukey’s fence-outliers.


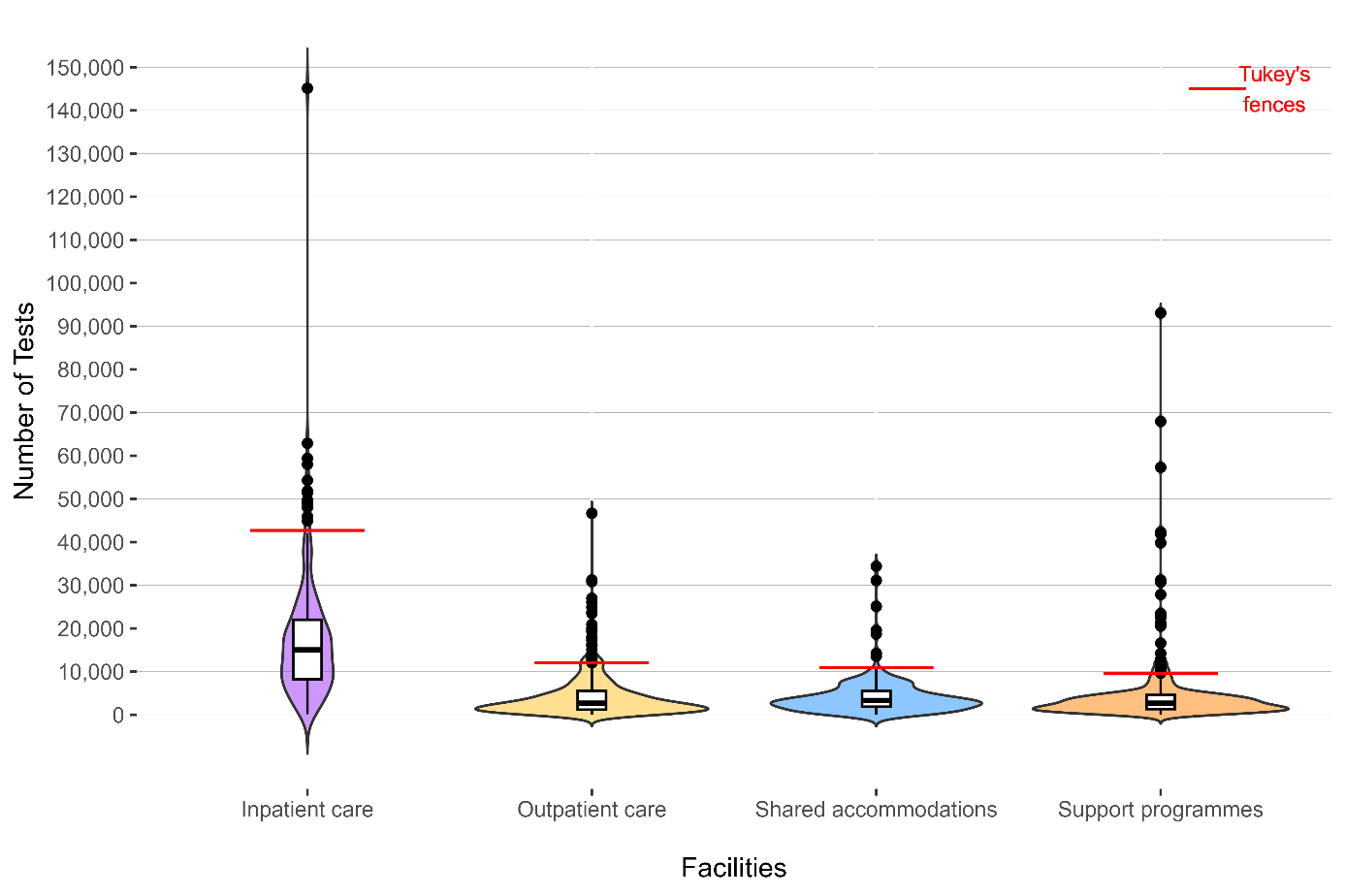


Supplemental Fig. 2 Number and rate of invalid RATs by facility category and ISO week from week 47 2020 to week 9 2023, with classification of COVID-19-waves in Germany by the RKI (Tolksdorf et al. 2021).


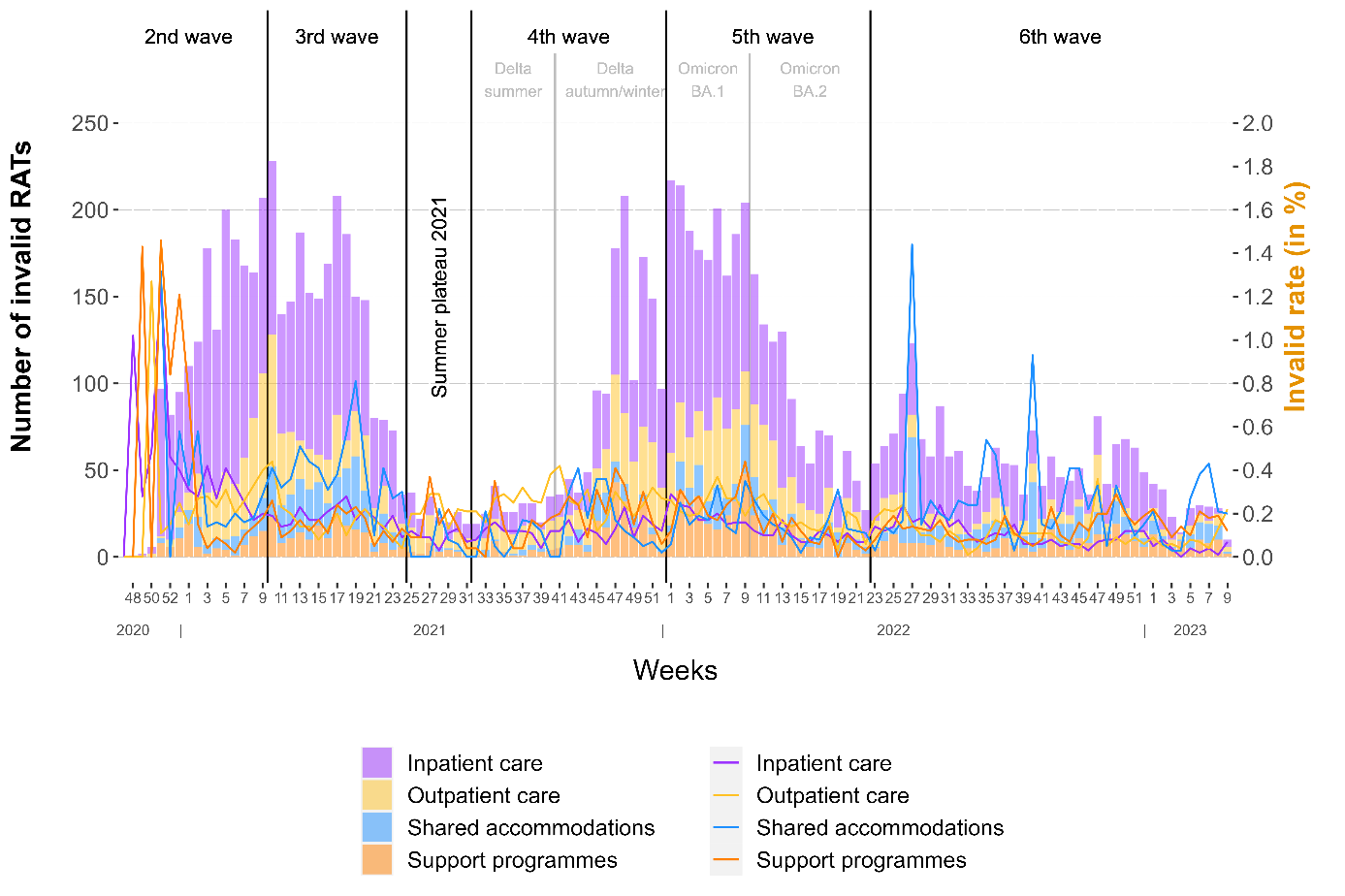


Supplemental Fig. 3. CROPS diagnostic of RATs and PCR cost degree of number of changepoints.


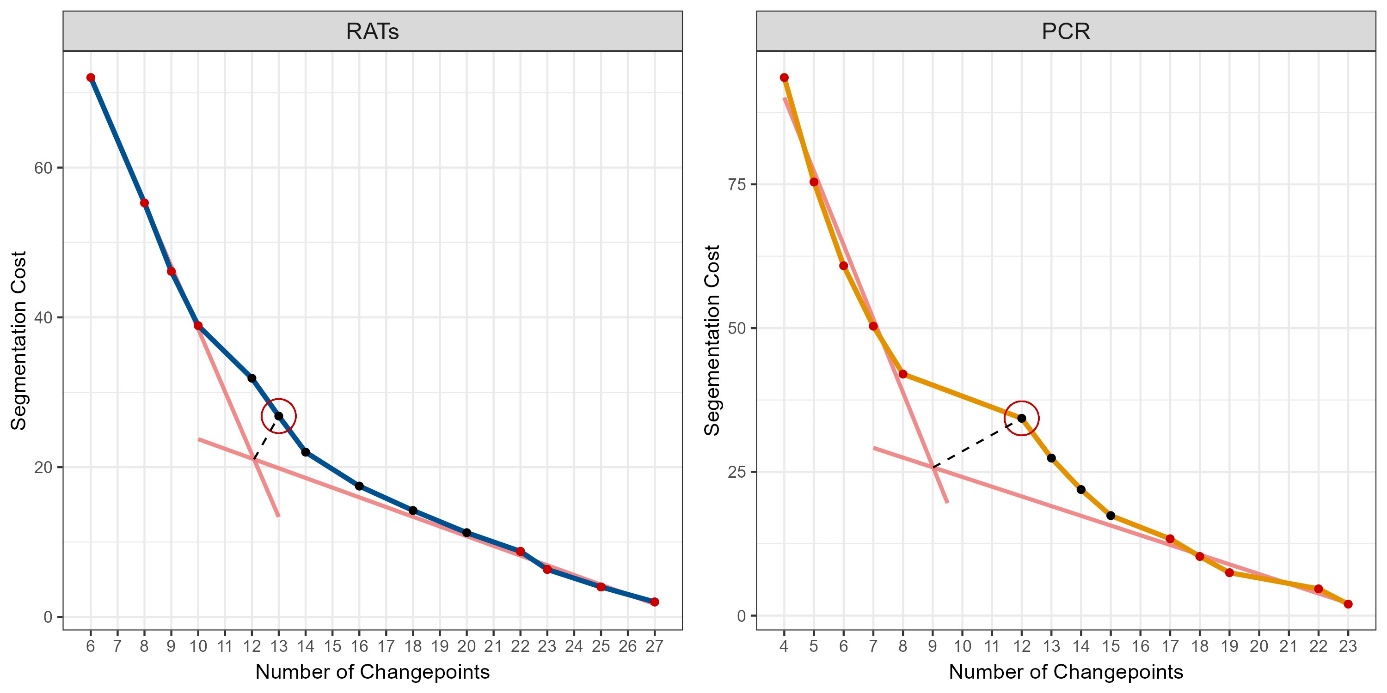


Supplemental Fig. 4 Weekly number differences of PCR and rapid antigen testing by ISO week from week 47 2020 to week 9 2023, with classification of COVID-19-waves in Germany by the RKI (Tolksdorf et al. 2021).


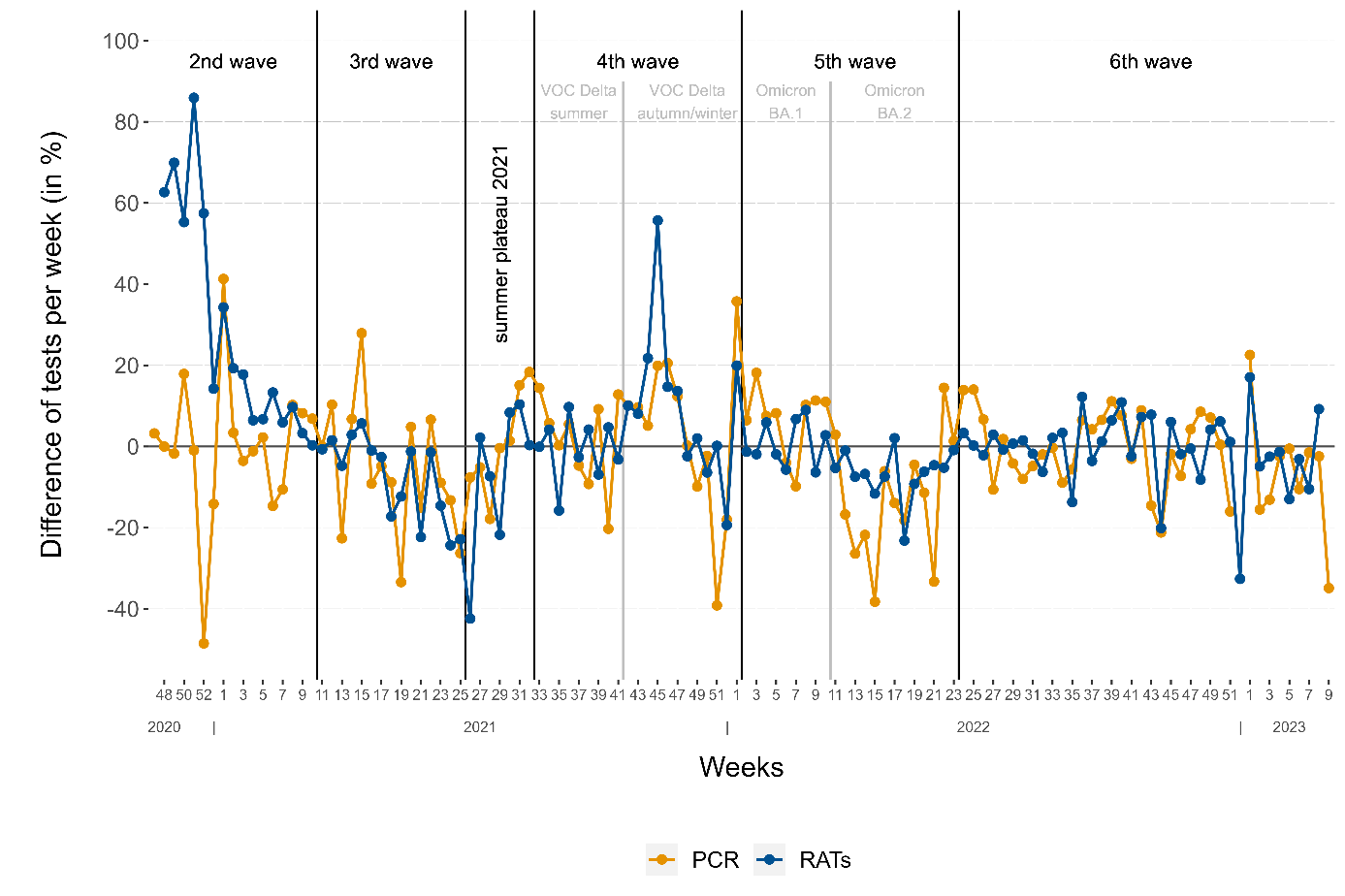


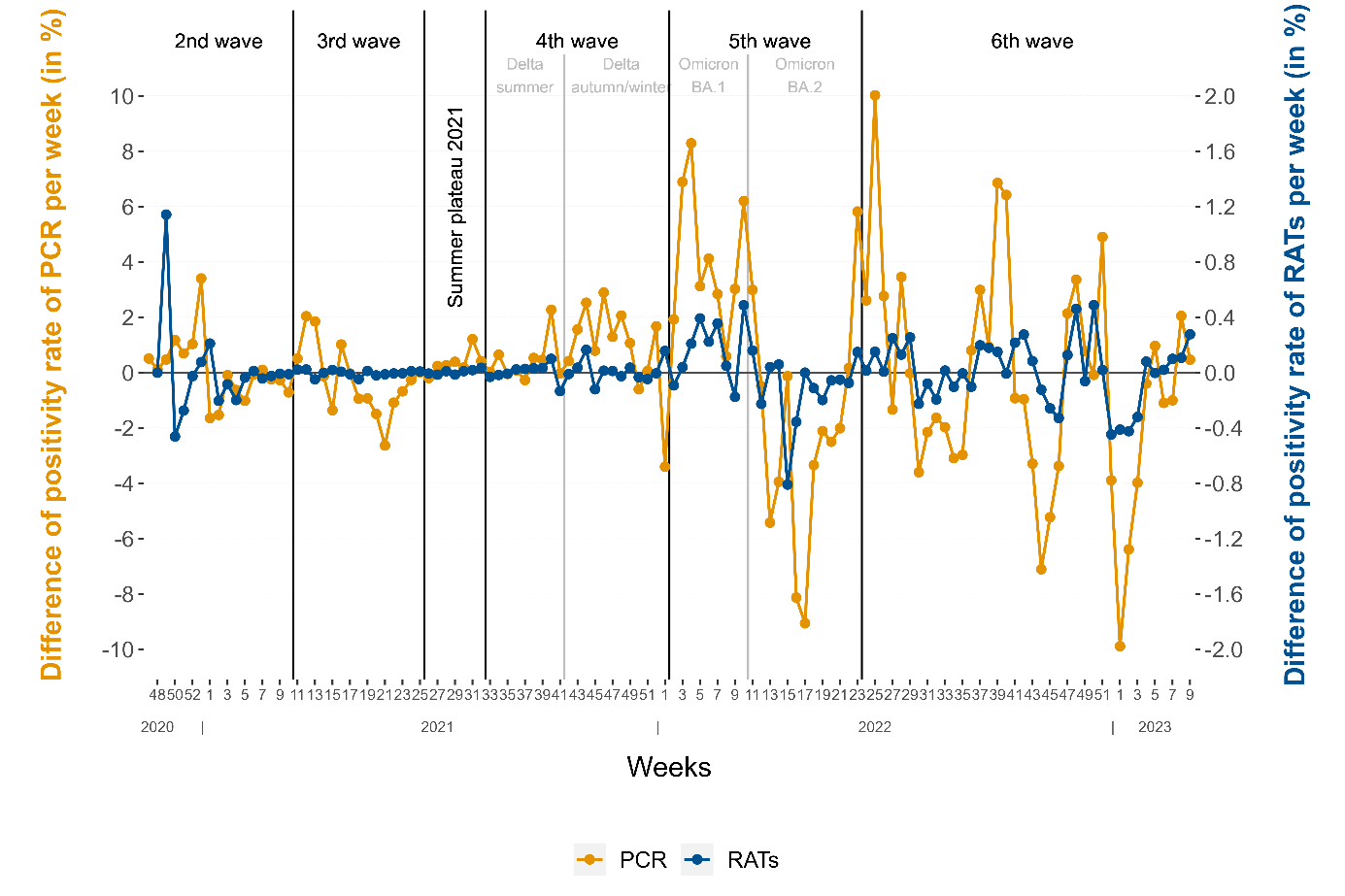


Supplemental Fig. 5 Weekly differences of the positivity rate of PCR and rapid antigen testing by ISO week from week 47 2020 to week 9 2023, with classification of COVID-19-waves in Germany by the RKI (Tolksdorf et al. 2021).

# Tables

Supplemental Table 1. Categories of COVID-19 rapid antigen testing reporting facilities.

| Main categories | Facility type |
| --- | --- |
| **Inpatient LTCF** | Inpatient LTCF |
| **Outpatient LTCF** | Outpatient LTCF |
|  | Offer of support in everyday life |
| **Shared accommodations** | Outpatient assisted living residencies |
|  | Special form of housing for integration assistance |
|  | Residential child care community |
|  | Emergency/ community accommodation |
| **Support programmes** | Other offer of the integration assistance |
|  | Day group |
|  | Day care |
|  | Workshop for disabled people |

Supplemental Table 2. Results of COVID-19 rapid antigen testing by facility category. (Abbreviations: CI 95=confidence interval 95 %, SD=standard deviation)

| Facility category (n) | Total (ISO week 47 2020 – week 9 2023) | | | | | | Weekly | | |
| --- | --- | --- | --- | --- | --- | --- | --- | --- | --- |
|  | **Reported weeks (total)** | **Total number of RATs used** | **Average number of**  **reported**  **RATs used (CI 95)**  **per facility** | **rate of negative RATs (n)** | **rate of positive RATs (n)** | **rate of invalid RATs (n)** | **Average number of reported**  **RATs used (CI 95) per facility** | **SD** | **Median** |
| Inpatient long-time care facilities (260) | 120 | 4,532,704 | 17,433.48 (15,668.68 - 19,198.27) | 99.24 (4,498,341) | 0.6 (27,085) | 0.16 (7,278) | 193.1 (175.73 - 210.47) | 142.24 | 162.02 |
| Outpatient long-time care services (391) | 120 | 1,771,845 | 4,531.57 (3,988.18 - 5,074.97) | 99.35 (1,760,341) | 0.51 (9,122) | 0.13 (2,382) | 57.09 (51.13 - 63.04) | 59.88 | 36.32 |
| Shared accommodations (150) | 117 | 663,641 | 4,424.27 (3,623.33 - 5,225.21) | 99.03 (657,209) | 0.73 (4,826) | 0.24 (1,606) | 50.33 (42.12 - 58.54) | 50.9 | 36.25 |
| Support programmes (292) | 120 | 1,438,818 | 4,927.46 (3,865.43 - 5,989.49) | 99.49 (1,431,503) | 0.32 (4,578) | 0.19 (2,737) | 60.08 (49.26 - 70.89) | 93.92 | 35.72 |
| **Total** (1093) | 120 | 8,407,008 | 7,691.68 (7,052.74 - 8,330.62) | 99.29 (8,347,394) | 0.54 (45,611) | 0.17 (14,003) | 85.98 (79.6 - 92.36) | 112.53 | 45.07 |
